# Identification of active regulatory regions from DNA methylation data

**DOI:** 10.1093/nar/gkt599

**Published:** 2013-07-04

**Authors:** Lukas Burger, Dimos Gaidatzis, Dirk Schübeler, Michael B. Stadler

**Affiliations:** ^1^Friedrich Miescher Institute for Biomedical Research, Maulbeerstrasse 66, 4058 Basel, Switzerland, ^2^Swiss Institute of Bioinformatics, Maulbeerstrasse 66, 4058 Basel, Switzerland and ^3^University of Basel, Petersplatz 1, 4003 Basel, Switzerland

## Abstract

We have recently shown that transcription factor binding leads to defined reduction in DNA methylation, allowing for the identification of active regulatory regions from high-resolution methylomes. Here, we present MethylSeekR, a computational tool to accurately identify such footprints from bisulfite-sequencing data. Applying our method to a large number of published human methylomes, we demonstrate its broad applicability and generalize our previous findings from a neuronal differentiation system to many cell types and tissues. MethylSeekR is available as an R package at www.bioconductor.org.

## INTRODUCTION

A critical step toward the understanding and modeling of mammalian gene regulation is the genome-wide and unbiased identification of regulatory regions. To this end, large efforts have been undertaken in recent years to map histone modifications as well as transcription factor-binding sites across many cell types and tissues (1–5). These studies have allowed cell type-specific annotation of regulatory regions such as active promoters and enhancers based on the presence of particular combinations of chromatin marks (6). DNA methylation has so far been mainly studied with a focus on CpG islands (CGIs) and was found to change comparatively little across cell types, thus containing little information about tissue-specific regulatory activity (7,8). However, using base-pair resolution whole-genome bisulfite sequencing (Bis-seq), we have recently shown in mouse embryonic stem cells (ESC) and neural progenitors (NP) that outside of CGIs, transcription factor binding leads to locally reduced DNA methylation levels in an otherwise fully methylated genome, allowing for the genome-wide identification of active and cell type-specific regulatory elements from Bis-seq data (9).

Here, we present MethylSeekR, a computational method for the identification of such footprints, implemented as an R/Bioconductor package. MethylSeekR builds on previously introduced ideas (9), but incorporates several methodological improvements and extensions that make it robust and generally applicable. The method is based on a cutoff approach that identifies hypomethylated regions as stretches of consecutive CpGs with methylation levels below a fixed threshold. To achieve high accuracy and sensitivity, MethylSeekR incorporates important preprocessing and filtering steps, and controls segmentation parameters via false discovery rate (FDR) calculations. Applying the method to a large number of human datasets (Supplementary Table S1), we show that MethylSeekR generally allows for the identification of active regulatory regions from Bis-seq data, thus generalizing our findings in mouse to many other cell types and tissues.

## MATERIALS AND METHODS

Datasets and annotations used are described in the Supplementary Material.

### FDR calculation

To calculate the FDR for a fixed cutoff on methylation *m* as well as on the minimal number of CpGs *n* per region, we compared the segmentation of the original methylome with the segmentation of a randomized methylome. To construct the randomized methylome, we randomly shuffled the methylation levels of all CpGs, destroying the spatial correlation of methylation levels between consecutive CpGs. The rationale of this approach is that due to noise one may encounter CpGs with reduced methylation, but these should not extensively cluster spatially. The FDR calculation is only relevant for regions containing few CpGs, as the likelihood of spatial clustering of CpGs with reduced methylation by chance decreases very rapidly with increasing numbers of CpGs. Because unmethylated regions (UMRs) contain by definition at least 30 CpGs, they are extremely unlikely to occur by chance, and it is therefore only the low-methylated regions (LMRs) that need to be assessed for their significance. Thus, for the randomization, we only used CpGs that do not overlap with CpG islands, which, if unmethylated, correspond to UMRs (we do not directly remove all CpGs overlapping UMRs, as it is undesirable to make the FDR calculation dependent on the segmentation of the original methylome, which also depends on *m* and *n*). To make sure that all unmethylated CpGs in UMRs overlapping CpG islands are removed, we extend all CpG islands to a total length of 5 kb.

### Segmentation of partially methylated domains

Partially methylated domains (PMDs) are characterized by highly disordered methylation, resulting in an average methylation clearly below the genomic background level (10,11). Because PMDs are generally large [mean length of 153 kb (10)], they do not need to be modeled at the single CpG level, but can be characterized using summary statistics in sliding windows containing several CpGs. Here, we choose windows of 101 CpGs (sliding one CpG at a time) and calculate a statistic that reflects the degree to which the distribution of methylation levels resembles a polarized distribution typically found in most mammalian datasets, which favors either low or high methylation levels, as in LMRs and UMRs, or the baseline methylation levels. In particular, we model the reads that cover each CpG as being generated from a beta binomial distribution (12), whereby, for a given CpG 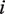, first the probability of it being methylated 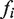 is sampled from a beta distribution,

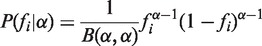

followed by a sampling of 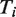 reads from a binomial model with the chosen probability 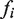,

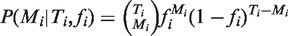




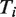
 is the total number of reads at CpG 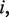 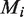 is the number of reads without a C-to-T conversion (indicating that the C was methylated) and 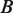 is the beta function, defined as

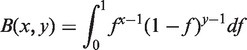



Here, we use a symmetric beta distribution characterized by a single parameter 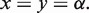 Distributions with 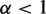 favor methylation levels that are polarized toward 0 and 100%; 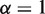
corresponds to a uniform distribution; and distributions with 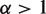 are biased toward intermediate methylation levels. If we assume that the methylation levels of all CpGs in a given window are drawn from the same beta distribution, defined by 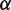, the total probability of the data (i.e. all the reads covering CpGs) in a window, assuming independence between the CpGs, can be written as

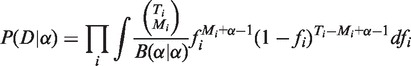

which evaluates to

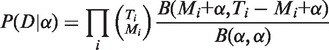

where the product runs over all CpGs *i* in the window, and 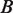 is the beta function. To characterize the distribution of methylation levels, we determine for each window the posterior mean of 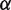. Because the integral over all 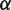
s in the expression for 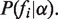 cannot be calculated analytically, we approximate the posterior by discretizing 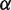 in bins of 0.1 from 0 to 3 and calculate the posterior mean as 
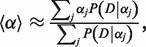
 where the 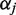
s correspond to the discretized values of 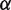 Inspection of the 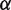 distribution in different methylomes reveals that most methylomes have a unimodal distribution with a mean clearly below 1, and a small number of methylomes (*imr90*, *ff*, *ads* and *ads_adipose*) have a bimodal or long-tailed distribution with a significant fraction of windows with 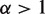, which are indicative of the presence of PMDs. For the latter methylomes, we trained a two-state Hidden Markov Model (HMM) with Gaussian emissions on the 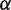 values via standard expectation maximization and predicted the location of PMDs using the Viterbi algorithm, as implement in the R package mhsmm (13). We first trained the HMM on the *imr90* methylome, using starting values of 0.5 and 1.5 for the means and 0.1 as the variance of the Gaussian distributions of the two states. For all other methylomes, we used the trained values of *imr90* as starting values. In a post-processing step, we first removed all predicted PMDs shorter than 101 CpGs and fused all PMDs separated by <101 CpGs, as the resolution of the approach is set by the window length of 101 CpGs.

### Motif enrichments

Prediction of transcription factor-binding sites and the calculation of motif enrichments were performed as in (9). Cell type-specific LMRs were defined as all LMRs that do not overlap with LMRs in any of the other methylomes, using a reduced set of methylomes including *h1*, *h1_bmp4*, *ads_adipose*, *imr90*, *hspc* and *bcell*. Constitutive LMRs were identified as follows: we determined all LMRs that overlapped with LMRs in at least two other methylomes (using the same reduced set of methylomes). Overlapping LMRs were fused, creating a new segment containing all the nucleotides of the original segments.

## RESULTS

**Figure 1.**
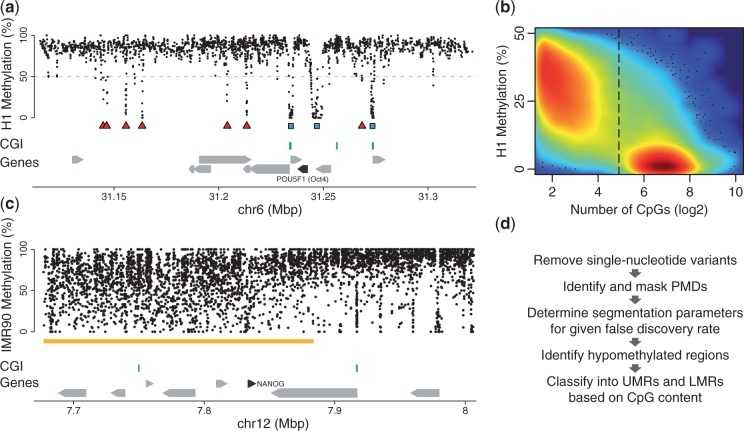
Identification of regulatory regions from Bis-seq data. (**a**) Typical methylation pattern in mammalian methylomes (dots represent individual CpGs, methylation levels averaged over three consecutive CpGs). UMRs (blue rectangles) and LMRs (red triangles) are identified as regions with methylation levels <50% (dashed gray line). CGI: CpG islands. (**b**) The number of CpGs per hypomethylated region versus its median methylation. The regions separate into two classes: CpG-rich, unmethylated UMRs and CpG-poor LMRs with residual methylation. (**c**) Some methylomes contain regions of highly disordered methylation (PMDs, orange bar, dots represent individual CpGs), which need to be identified and masked for the identification of regulatory regions. Unsmoothed methylation levels are shown. (**d**) Workflow of MethylSeekR.

The typical CpG methylation pattern in mammalian genomes (here H1 human ESCs) is shown in Figure 1a. Most of the genome is fully methylated, sporadically interspersed by short regions with reduced methylation, evident as stripes in the profile. We have previously shown that these regions belong to one of two distinct classes: CpG-rich, completely unmethylated (UMRs, blue rectangles) and CpG-poor, low-methylated regions (LMRs, red triangles), corresponding to proximal and distal regulatory sites, respectively (9). The basic principle of MethylSeekR is to identify hypomethylated regions by determining stretches of consecutive CpGs with methylation levels below a fixed cutoff (*m*) containing a minimal number of CpGs (*n*). The identified regions are then further classified as UMRs or LMRs based on their CpG content. To achieve high accuracy and sensitivity, the approach has to take into account the occurrence of single-nucleotide variants (SNVs) as well as the statistical sampling noise at individual CpGs. In addition, it needs to estimate an FDR for the choice of appropriate values for *n* and *m*, and has to be able to differentiate UMRs and LMRs from PMDs (10).

### Filtering of SNVs

In Bis-seq experiments, methylation levels of individual CpGs are inferred as the fraction of aligned reads without a cytosine (C) to thymine (T) mismatch (14). In this context, SNVs that differ between the genome sequence under study and the reference genome require special attention (15). Cs in CpG context are hot-spots for sequence variation (16) and, when mutated, can lead to incorrect estimation of methylation levels. For example, a heterozygous locus might be wrongly classified as partially methylated, and a homozygous locus even as unmethylated. The underlying genetic differences, namely C-to-T mutations, cannot be discriminated from the bisulfite-induced conversion of unmethylated Cs. This problem can be solved if the genome sequence of the experimental system is known; either obtained by genome sequencing or, in the case of high coverage methylomes, by examination of the mismatch pattern in alignments to the G on the opposite strand of the CpG under consideration. Mismatches at the G at a higher frequency than expected due to sequencing errors are indicative of single nucleotide variations and allow identification of the problematic Cs (17). In the set of published human methylomes analyzed here (Supplementary Table S1), since we do not know the genetic background of the analyzed cell types and due to the limited coverage, we at least partially circumvent this problem by removing all CpGs that overlap with SNVs from dbSNP (18) (Supplementary Figure S1).

### Data preprocessing

The accuracy of the estimated methylation levels at individual CpGs is strongly dependent on the total number of aligned reads. Many published methylomes have sequencing depths of around 10-fold and thus a substantial uncertainty in methylation estimates at individual CpGs, in particular at CpGs with intermediate methylation levels (Supplementary Figures S2 and S3). This problem can be mitigated by averaging methylation levels over a fixed number of consecutive CpGs (19). We have previously shown that most hypomethylated regions overlap with DNase I hypersensitive sites (DHSs) (9) that are indicative of transcription factor-binding sites (20). A comparison of DHSs with hypomethylated regions as a function of the number of CpGs they contain reveals that, given the coverage constraints of typical published datasets, three CpGs is the lowest resolution at which regulatory regions can be inferred with high accuracy (Supplementary Figure S4). In the analyses presented here and as a default in MethylSeekR, we thus smooth methylation levels over three consecutive CpGs, demanding a minimal coverage of 5 reads per CpG. This results in a methylation read-out for 78–94% of CpGs in the datasets analyzed in this study (see below).

### Identification of hypomethylated regions

After removal of SNVs and smoothing, hypomethylated regions are identified as stretches of CpGs with methylation levels below a user-defined cutoff *m* containing a minimal number of *n* CpGs*. m* and *n* are crucial parameters that strongly affect the segmentation results. To choose suitable parameter values, we estimate for each methylome an FDR, defined by the number of identified segments in the original methylome relative to the ones identified in a randomized methylome, in which the methylation levels of the CpGs have been shuffled (9,12). The randomization destroys the correlation of methylation levels between neighboring CpGs and is used to assess the frequency of spatial clustering of hypomethylated CpGs by chance (Materials and Methods). The relationship between FDR, the number of identified regions as well as *m* and *n* is shown in Supplementary Figure S5. It shows that there is a trade-off between the two parameters, allowing for similar results through various combinations of *m* and *n.* Furthermore, it illustrates important differences between methylomes. Some methylomes display larger variability of methylation levels and in turn require more stringent parameter settings (see below). For the analyses presented here, we set *m* to 50% and choose the smallest *n* that results in an FDR <5%. With this choice of parameters, DHSs in both mouse and human ESCs are recovered with high accuracy and good sensitivity (Supplementary Figure S6).

### Classification of hypomethylated regions into UMRs and LMRs

Plotting median methylation levels against the number of CpGs per identified region (Figure 1b, Supplementary Figure S7) reveals a striking separation of the hypomethylated regions into two classes, a class of CpG-rich and unmethylated regions and a second one of CpG-poor regions with low methylation levels between 10 and 50%, which correspond to the previously identified UMRs and LMRs, respectively (9). The two classes of segments differ in both methylation levels and CpG content. Because CpG content more clearly distinguishes the two classes (Supplementary Figure S7), it is used to separate the identified regions into UMRs and LMRs, at a cutoff of 30 CpGs (dashed line in Figure 1b).

### Identification and masking of PMDs

In some methylomes, the typical methylation pattern (Figure 1a) is interrupted by regions of highly disordered methylation (Figure 1c). Owing to their reduced average methylation levels, these regions were termed PMDs and shown to overlap with genomic regions that are in a transcriptionally repressed state (10). Because PMDs can cover up to 40% of the genome (10,11) and have heterogeneous methylation levels, they contain a large number of CpGs with reduced methylation levels that would erroneously be classified as LMRs or UMRs. Therefore, they need to be accurately identified and masked at the beginning of the analysis. To this end, we developed an HMM, which considers sliding windows of 100 consecutive CpGs and classifies them based on the shape of the distribution of methylation levels. In particular, the HMM identifies PMDs by the divergence of their methylation level distributions from the typical polarized distribution, which favors high and low methylation as in the fully methylated baseline methylation and UMRs or LMRs, respectively (Materials and Methods).

### MethylSeekR workflow

**Figure 2.**
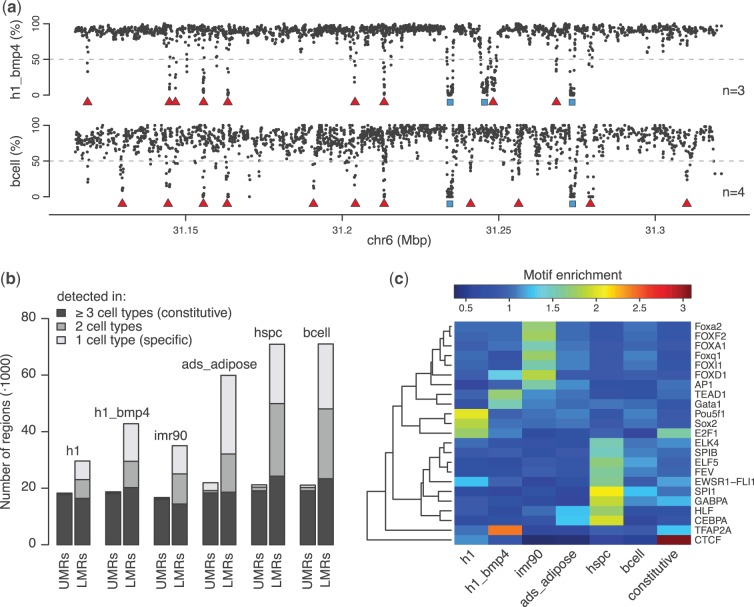
LMRs are highly dynamic distal regulatory elements. (**a**) Methylation profiles for trophoblasts differentiated from H1 (h1_bmp4) and B cells (bcell) for the same locus as in Figure 1a. UMRs and LMRs are shown as blue rectangles and red triangles, respectively, and n indicates the minimal number of CpGs required to identify a region. (**b**) Number of UMRs and LMRs identified in selected human methylomes: H1 ESCs (h1), trophoblasts differentiated from H1 (h1_bmp4), fetal lung fibroblasts (imr90), adipocytes differentiated from adipose-derived stem cells (ads_adipose), hematopoietic stem and progenitor cells (hspc) and B cells (bcell). The regions have been grouped by the number of cell types they exist in. (**c**) Transcription factor motif enrichments for cell type-specific and constitutive LMRs. Only motifs with an enrichment >1.5 in at least one cell type are shown.

The complete workflow of MethylSeekR is summarized in Figure 1d: CpGs overlapping SNVs are removed, and PMDs are identified and masked. After smoothing of methylation levels, the algorithm calculates the FDR for various combinations of *m* and *n* as a guide to select appropriate segmentation parameters. Finally, the algorithm provides a list of all hypomethylated regions classified into UMRs and LMRs. These regions can furthermore be used as the basis for a differential analysis comparing two or more methylomes (Figure 2).

### Sequencing depth requirements

An important step in Bis-seq experiments is to determine the average read coverage required to identify regions of interest. To investigate to what extent the identification of UMRs and LMRs with MethylSeekR depends on the average coverage of the Bis-seq sample, we applied the method to sub-sampled methylomes of two datasets with coverage >30-fold and compared the identified regions with the regions obtained from the full-coverage methylomes (Supplementary Figure S8). This analysis revealed that due to the stringency of our parameter settings, in particular the requirement to use only CpGs with coverage of at least 5 reads for segmentation, regions are identified at high accuracy for an average genome-wide coverage as low as 5-fold. However, lowering the coverage comes at the price of a decreased sensitivity, in particular a loss of short LMRs. Whereas a large fraction of UMRs can be detected at a mean coverage as low as 5, a coverage of at least 10-fold is required to identify the majority of LMRs (∼80%). A subsampling analysis can only be used as a rough guide to estimate coverage requirements, as it cannot take into account potential experiment-specific sources of variation, such as varying coverage distributions and noise levels. Nevertheless, we believe that an average genome-wide coverage of at least 10-fold is a good starting point for any high-resolution methylome analysis. If SNVs need to be detected from the same data, a coverage as high as 30-fold is recommended (17).

### Application of MethylSeekR to published human methylomes

To our knowledge, MethylSeekR is the only available software that identifies UMRs and LMRs from genome-wide Bis-seq datasets and can therefore not be compared with existing methods. We validated the accuracy and robustness of MethylSeekR in two ways. Firstly, we compared the identified regions with DHSs, which are commonly used as a gold standard for transcription factor binding. This analysis revealed that the large majority of identified regions overlap DHSs (Supplementary Figure S6). Secondly, we applied MethylSeekR to a large number of published human methylomes (10,11,21,22). These include methylomes of human ESCs, induced pluripotent (iPS) cells, fibroblasts, adipose tissue and cell types of the hematopoietic lineage (Supplementary Table S1, segmentation examples for a representative set of methylomes can be downloaded from *www.fmi.ch/groups/gbioinfo*). These datasets are diverse in terms of cell type, coverage (Supplementary Figure S2), noise level and presence or absence of PMDs. For example, Figure 2a shows representative methylation profiles for trophoblasts differentiated from H1 (h1_bmp4) and for B cells (bcell). Clearly, the B-cell methylome shows much more variability in background methylation levels than the trophoblast methylome. In accordance with this, a larger minimal number of CpGs per hypomethylated region is required to keep the FDR <5%. Importantly, whereas for our previously analyzed mouse ESC and NP methylomes (9), which are devoid of PMDs and have low noise levels, MethylSeekR identifies a highly similar set of regions as our previously proposed method, many of these human methylomes would have been difficult to analyze using our previous approach (see Supplementary Material for an in-depth discussion).

In accordance with previous studies (10,11), we found clear evidence for PMDs in four methylomes (IMR90, foreskin fibroblasts, adipose-derived stem cells and adipocytes, Supplementary Figure S9). After filtering of PMDs, we identified between 50 000 and 100 000 hypomethylated regions per methylome, with a stable number of UMRs and a larger more variable number of LMRs (Figure 2b, Supplementary Figure S10, identified PMDs, UMRs and LMRs can be downloaded from *www.fmi.ch/groups/gbioinfo*). A comparison of the identified regions in ESCs and iPS cells demonstrated good reproducibility and showed that both UMRs and LMRs are conserved in pluripotent cells (Supplementary Figure S11). Overlapping hypomethylated regions with genomic annotations revealed that UMRs correspond mostly to promoters, while LMRs lie in intergenic or intronic regions distal to transcription start sites (Supplementary Figures S12 and S13). Sequence conservation analysis revealed that both UMRs and LMRs are more conserved than their surrounding regions (Supplementary Figure S14), suggesting that they represent regulatory regions. In accordance with our previous work, UMRs are mostly stable across tissues (Figure 2b, Supplementary Figure S15), whereas LMRs are highly dynamic (Figure 2b, Supplementary Figure S16).

To further characterize LMRs, we performed a motif enrichment analysis using 130 weight matrices from the Jaspar database (23). In accordance with our previous findings (9), this revealed enrichments for cell type-specific transcription factor (TF) motifs in cell type-specific LMRs, such as Pou5f1 and Sox2 in H1 ESCs (24), AP-2alpha (TFAP2A) in trophoblasts (h1_bmp4) (25), C/EBP-alpha (CEBPA) in adipocytes (26), PU.1 (SPI1) in the hematopoietic lineage (27), and enrichment for constitutive TFs such as CTCF in constitutive LMRs (Figure 2c).

## DISCUSSION

We here present MethylSeekR, a computational method for the robust identification of regulatory regions from Bis-seq data. MethylSeekR takes as input a table with genomic coordinates and methylation states for individual CpGs and an optional table with known SNVs. It then filters CpGs overlapping SNVs, identifies and masks PMDs, calculates FDRs that allow a straightforward setting of segmentation parameters and finally identifies both proximal and distal regulatory regions (Figure 1d). The algorithm is implemented in an easy-to-use and fully documented R package that describes in detail each step of the analysis and produces several control plots (Supplementary Figures S5, S7 and S9) to facilitate the interpretation of the results and to avoid potential pitfalls in the analysis.

By analyzing a large number of published human methylomes, we demonstrate that MethylSeekR reliably identifies UMRs and LMRs, corresponding to proximal and distal regulatory regions, across many cell types and tissues, irrespective of the presence of PMDs or differences in noise levels. While regulatory regions can also be identified on the basis of DNaseI hypersensitivity or histone modifications, measuring DNA methylation is experimentally easier and does not require such high amounts of fresh starting material. For the study of rare cell types, DNA methylation profiling and analysis may thus currently be the only feasible approach for the experimental identification of regulatory regions. We believe that our method will greatly facilitate the analysis of such datasets and will make Bis-seq data a valuable source for the identification of active regulatory regions.

## SUPPLEMENTARY DATA

Supplementary Data are available at NAR Online, including [28,29].

## FUNDING

Novartis Research Foundation; Swiss initiative in Systems Biology (Cell Plasticity) (to research in the group of M.S.). Funding for open access charge: Institutional funds of the Friedrich Miescher Insitute for Biomedical Research.

*Conflict of interest statement*. None declared.

## Supplementary Material

Supplementary Data

## References

[1] Birney E, Stamatoyannopoulos JA, Dutta A, Guigo R, Gingeras TR, Margulies EH, Weng Z, Snyder M, Dermitzakis ET, Thurman RE (2007). Identification and analysis of functional elements in 1% of the human genome by the ENCODE pilot project. Nature.

[2] Bernstein BE, Stamatoyannopoulos JA, Costello JF, Ren B, Milosavljevic A, Meissner A, Kellis M, Marra MA, Beaudet AL, Ecker JR (2010). The NIH roadmap epigenomics mapping consortium. Nat. Biotechnol..

[3] Dunham I, Kundaje A, Aldred SF, Collins PJ, Davis CA, Doyle F, Epstein CB, Frietze S, Harrow J, Kaul R (2012). An integrated encyclopedia of DNA elements in the human genome. Nature.

[4] Shen Y, Yue F, McCleary DF, Ye Z, Edsall L, Kuan S, Wagner U, Dixon J, Lee L, Lobanenkov VV (2012). A map of the cis-regulatory sequences in the mouse genome. Nature.

[5] Zhu J, Adli M, Zou JY, Verstappen G, Coyne M, Zhang X, Durham T, Miri M, Deshpande V, De Jager PL (2013). Genome-wide chromatin state transitions associated with developmental and environmental cues. Cell.

[6] Ernst J, Kellis M (2010). Discovery and characterization of chromatin states for systematic annotation of the human genome. Nat. Biotechnol..

[7] Mohn F, Weber M, Rebhan M, Roloff TC, Richter J, Stadler MB, Bibel M, Schubeler D (2008). Lineage-specific polycomb targets and de novo DNA methylation define restriction and potential of neuronal progenitors. Mol. Cell.

[8] Bock C, Beerman I, Lien WH, Smith ZD, Gu H, Boyle P, Gnirke A, Fuchs E, Rossi DJ, Meissner A (2012). DNA methylation dynamics during in vivo differentiation of blood and skin stem cells. Mol. Cell.

[9] Stadler MB, Murr R, Burger L, Ivanek R, Lienert F, Scholer A, van Nimwegen E, Wirbelauer C, Oakeley EJ, Gaidatzis D (2011). DNA-binding factors shape the mouse methylome at distal regulatory regions. Nature.

[10] Lister R, Pelizzola M, Dowen RH, Hawkins RD, Hon G, Tonti-Filippini J, Nery JR, Lee L, Ye Z, Ngo QM (2009). Human DNA methylomes at base resolution show widespread epigenomic differences. Nature.

[11] Lister R, Pelizzola M, Kida YS, Hawkins RD, Nery JR, Hon G, Antosiewicz-Bourget J, O'Malley R, Castanon R, Klugman S (2011). Hotspots of aberrant epigenomic reprogramming in human induced pluripotent stem cells. Nature.

[12] Molaro A, Hodges E, Fang F, Song Q, McCombie WR, Hannon GJ, Smith AD (2011). Sperm methylation profiles reveal features of epigenetic inheritance and evolution in primates. Cell.

[13] O'connell J, Hojsgaard S (2011). Hidden semi Markov models for multiple observation sequences: the mhsmm package for R. J. Stat. Softw..

[14] Frommer M, McDonald LE, Millar DS, Collis CM, Watt F, Grigg GW, Molloy PL, Paul CL (1992). A genomic sequencing protocol that yields a positive display of 5-methylcytosine residues in individual DNA strands. Proc. Natl Acad. Sci. USA.

[15] Krueger F, Kreck B, Franke A, Andrews SR (2012). DNA methylome analysis using short bisulfite sequencing data. Nat. Methods.

[16] Cooper DN, Krawczak M (1989). Cytosine methylation and the fate of CpG dinucleotides in vertebrate genomes. Hum. Genet..

[17] Liu Y, Siegmund KD, Laird PW, Berman BP (2012). Bis-SNP: combined DNA methylation and SNP calling for Bisulfite-seq data. Genome Biol..

[18] Sherry ST, Ward MH, Kholodov M, Baker J, Phan L, Smigielski EM, Sirotkin K (2001). dbSNP: the NCBI database of genetic variation. Nucleic Acids Res..

[19] Hansen KD, Timp W, Bravo HC, Sabunciyan S, Langmead B, McDonald OG, Wen B, Wu H, Liu Y, Diep D (2011). Increased methylation variation in epigenetic domains across cancer types. Nat. Genet..

[20] Crawford GE, Holt IE, Mullikin JC, Tai D, Blakesley R, Bouffard G, Young A, Masiello C, Green ED, Wolfsberg TG (2004). Identifying gene regulatory elements by genome-wide recovery of DNase hypersensitive sites. Proc. Natl Acad. Sci. USA.

[21] Laurent L, Wong E, Li G, Huynh T, Tsirigos A, Ong CT, Low HM, Kin Sung KW, Rigoutsos I, Loring J (2010). Dynamic changes in the human methylome during differentiation. Genome Res..

[22] Hodges E, Molaro A, Dos Santos CO, Thekkat P, Song Q, Uren PJ, Park J, Butler J, Rafii S, McCombie WR (2011). Directional DNA methylation changes and complex intermediate states accompany lineage specificity in the adult hematopoietic compartment. Mol. Cell.

[23] Bryne JC, Valen E, Tang MH, Marstrand T, Winther O, da Piedade I, Krogh A, Lenhard B, Sandelin A (2008). JASPAR, the open access database of transcription factor-binding profiles: new content and tools in the 2008 update. Nucleic Acids Res..

[24] Chambers I, Tomlinson SR (2009). The transcriptional foundation of pluripotency. Development.

[25] Cheng YH, Aronow BJ, Hossain S, Trapnell B, Kong S, Handwerger S (2004). Critical role for transcription factor AP-2alpha in human trophoblast differentiation. Physiol. Genomics.

[26] Lefterova MI, Zhang Y, Steger DJ, Schupp M, Schug J, Cristancho A, Feng D, Zhuo D, Stoeckert CJ, Liu XS (2008). PPARgamma and C/EBP factors orchestrate adipocyte biology via adjacent binding on a genome-wide scale. Genes Dev..

[27] Scott EW, Simon MC, Anastasi J, Singh H (1994). Requirement of transcription factor PU.1 in the development of multiple hematopoietic lineages. Science.

[28] Meyer LR, Zweig AS, Hinrichs AS, Karolchik D, Kuhn RM, Wong M, Sloan CA, Rosenbloom KR, Roe G, Rhead B (2013). The UCSC Genome Browser database: extensions and updates 2013. Nucleic Acids Res..

[29] Lawrence M, Gentleman R, Carey V (2009). rtracklayer: an R package for interfacing with genome browsers. Bioinformatics.

