# Supplementary material for: Identification of active regulatory regions from DNA methylation data
Source: Nucleic Acids Res. 2013 Jul 4;41(16):e155. doi: 10.1093/nar/gkt599 (PMC3763559; doi:10.1093/nar/gkt599)
Supplement: Supplementary Data [file supp_41_16_e155__index.html]

Identification of active regulatory regions from DNA methylation data — Identification of active regulatory regions from DNA methylation data — Supplementary Data 

# Identification of active regulatory regions from DNA methylation data

## 

files

**Files in this Data Supplement:**

- Supplementary Data - pdf file
