# Supplementary material for: Identification of active regulatory regions from DNA methylation data
Source: Nucleic Acids Res. 2013 Jul 4;41(16):e155. doi: 10.1093/nar/gkt599 (PMC3763559; doi:10.1093/nar/gkt599)
Supplement: Supplementary Data [file supp_gkt599_nar-00704-met-g-2013-File004.pdf]

1 Datasets and annotation

All analyses were done using the hg18 assembly for human and the mm9 assembly for mouse. The *h1, h9, h9_laurent, imr90_ipsc, ff_ipsc_19_11, ff_ipsc_19_7, ff_ipsc_6_9, ads_ipsc, h1_bmp4, ff_ipsc_bmp4, iPSCs, ads, ads_adipose, imr90* and *ff* methylomes were downloaded from *http://neomorph.salk.edu/ips_methylomes/data.html* . The *hspc, cd133hsc, neutrophil* and *bcell* methylomes were downloaded from GEO (accessions GSM791828, GSM791830, GSM791829 and GSM791827). The methylome data for mouse embryonic stem cells and neural progenitors can be found on GEO, accession GSE30206.

For mouse, single-nucleotide variations (SNVs) were defined as described in [1]. For human, SNVs were downloaded from dbSNP [2] (*ftp://ftp.ncbi.nih.gov/snp/organisms/human_9606/chr_rpts/* , downloaded on March 22, 2010, release B132) for hg19 and lifted to hg18 using the liftOver tool from UCSC [3].

DnaseI hypersensitive sites were downloaded from ENCODE (*http://genome.ucsc.edu/ENCODE/* [4], two replicates for mouse embryonic stem cells, wgEncodeUwDNaseIEscj7S129ME0HotspotsRep1.broadPeak and wgEncodeUwDNaseIEscj7S129ME0HotspotsRep2.broadPeak and one replicate for human H1 cells, wgEncodeUwDNaseISeqRawDataRep1H1es.broadPeak). We only used sites with a p-value below 10^{-3}. In the case of mouse, we only retained the sites that overlapped between the two replicates and fused them into longer sites containing all nucleotides of the original sites.

Genomic regions were defined as in [1] using the annotation of known RefSeq transcripts from UCSC (*http://hgdownload.cse.ucsc.edu/goldenPath/hg18/database/refGene.txt.gz* , downloaded on August 13, 2012 and *http://hgdownload.cse.ucsc.edu/goldenPath/mm9/database/refGene.txt.gz* , downloaded on August 21, 2012). CpG island coordinates were downloaded from UCSC using the R package rtracklayer ([5], "cpgIslandExt" table). For conservation analysis in human, we used the PhastCons track phastCons44way from UCSC (*http://genome.ucsc.edu/* , downloaded on Nov 4, 2010). The datasets used for **Supplementary Figure 17** are described in [1].

2 Comparison of MethylSeekR with previously proposed approach

For the discovery of UMRs and LMRs in mouse embryonic stem cell (ESC) and neural progenitor (NP) methylomes, we have previoulsy proposed a three-state Hidden Markov Model (from here on referred to as the HMM model) [1]. Briefly, in this model, the three states correspond to a fully-methylated state which emits background methylation levels around 80%, a low-methylated state which emits methylation levels around 30% and an unmethylated state that emits methylation levels around 0%. Regions in the low-methylated state correspond to LMRs and regions in the unmethylated state to UMRs.

To directly compare MethylSeekR with this previously proposed approach, we looked at the overlap of the identified regions (UMRs and LMRs) in mouse ESCs and NPs when using $m = 50\%$ and $n = 3$ (which corresponds to the smallest *n* such that the FDR $< 5\%$) for MethylSeekR. Importantly, we cannot directly compare MethylSeekR to the HMM model on the human methylomes analysed in this study, as the HMM model does not include functionality to take into account differing amounts of noise nor to mask partially methylated domains (see below).

In ESCs/NPs, 89/90% of the regions identified by MethylSeekR overlap (by at least one nucleotide) with the regions identified by the HMM and 96/98% of the regions identified by the HMM overlap with the segments identified by MethylSeekR. On the level of single CpGs, 95/96% of the CpGs in the MethylSeekR regions overlap with CpGs in the HMM-derived regions and 98/99% of CpGs in the HMM-derived regions overlap with the MethylSeekR regions. Outside of CpG islands, these numbers decrease to 94/94% and 86/87%. This shows that there is a very good agreement between the two methods. Generally, the MethylSeekR segmentation results in a smaller number of segments (n=55059/34746 versus n=60010/48769). This difference is mostly due to differences in the segmentation of unmethylated CpG islands. The MethylSeekR segmentation is not affected by moderately elevated methylation levels at a few CpGs within otherwise unmethylated CpG islands (which occur quite frequently, particularly in NPs), whereas the HMM is sensitive to such variations and tends to cut these CpG islands into smaller pieces, resulting in several UMRs compared to one single UMR (data not shown).

We believe that MethylSeekR has, in addition to its simplicity and computational efficiency, several

important advantages over the HMM approach. First, only MethylSeekR can identify and filter partially methylated domains, which is a crucial and indispensable step in the identification of regulatory regions in PMD-containing methylomes. PMDs can cover up to 40% of the genome [6, 7] and due to the heterogeneity in methylation levels, they contain a very large number of CpGs with reduced methylation levels that would wrongly be classified as UMRs or LMRs.

Second, with regard to the segmentation of UMRs and LMRs, the method has fewer free parameters (only m, n, cut-off on the coverage and smoothing kernel width) and we show that the most important of these, m and n, can be determined via a FDR calculation scheme. The HMM on the other hand has many more parameters, such as transition and emission parameters, which can be trained via standard expectation-maximization, but need to be manually adjusted to reduce the FDR [1] and thus cannot easily (unlike MethylSeekR) be operated in an unsupervised manner.

Third, whereas MethylSeekR is free of parametric assumptions, the HMM makes assumptions about the shape of emission distributions as well as the length-distribution of the hypomethylated regions. In particular, the model structure implies a geometric length distribution in the number of CpGs, which is violated in the case of UMRs. Thus, even though there is a fairly clear separation in median methylation levels between LMRs and UMRs, the HMM cannot perform a clean separation of the two classes and tends to classify many regions with few CpGs as UMRs, even if they have residual methylation levels ([1], data not shown).

Fourth, the HMM model separates UMRs and LMRs mainly via their difference in methylation levels. However, we believe that using the number of CpGs instead of the methylation levels for the classification of UMRs and LMRs is a more robust approach because the number of CpGs in a given region is fixed and determined by the DNA sequence whereas methylation levels can fluctuate due to variations in conversion efficiency, coverage, global changes in methylation levels as well as variability between cell types. This can be appreciated in **Supplementary Figure 7**, in which the number of CpGs per region is plotted against median methylation for all regions identified in the different methylomes. Whereas the average methylation in UMRs and LMRs globally change from methylome to methylome, with varying bimodality, the separation in the number of CpGs is always strong and stays roughly constant, allowing the use of a fixed cut-off for the classification.

Due to the imprecise separation of UMRs and LMRs because of the varying bimodality in methylation levels and the geometric length distribution constraint of the HMM model, the classification of regions into UMRs and LMRs via the number of CpGs per regions (as used in MethylSeekR) leads to a reassignment of hypomethylated regions to UMRs and LMRs. In the case of our previously published methylomes (mouse ESC and NP), this leads to a reclassification of 10632/5567 UMRs (as defined by the HMM) to LMRs (in contrast to the small number of LMRs reclassified as UMRs (1788/1076)). These novel LMRs have the same enrichment for enhancer chromatin marks as the LMRs identified by the HMM (**Supplementary Figure 17**) and additionally, the reclassification leads to a much clearer separation of UMRs and LMRs into proximal and distal regulatory elements (**Supplementary Figure 18**). These findings support the validity of the reclassification by MethylSeekR, leaving our previous conclusions [1] unchanged.

Finally and importantly, unlike our previous HMM approach, the MethylSeekR software is publicly available and we provide it as an easy-to-use and fully documented R package, which includes a detailed description of the analysis steps for an example methylome and should thus greatly facilitate the identification of regulatory regions from Bis-seq data.

3 Subsampling of methylomes

Starting with a table of counts for each CpG, we subsampled methylomes as follows. We considered each count in the table as a separate DNA molecule that is either methylated or unmethylated. We then picked from the pool of all molecules (representing all counts of all CpGs in the table) a total of $n = c * n_{tot}$ molecules at random without replacement, where n_{tot} is total number of CpGs in the genome and c is the desired average coverage. Counting these molecules results in a subsampled table, which we then used to identify UMRs and LMRs using MethylSeekR.

4 Comparison of methylomes on region level

For the comparison of methylation levels on the level of regions (**Supplementary Figure 11**, **Supplementary Figure 15** and **Supplementary Figure 16**), we defined, for each pair-wise comparison, the joint set of regions as the union of all regions of both methylomes. For comparisons involving PMD-containing methylomes, only the regions that did not overlap with PMDs in any of the two cell types were used. If two (or several) regions overlapped, they were fused into one larger region that contained all the nucleotides of the original regions. For both methylomes, methylation levels were then calculated for the joint set of regions to produce the final scatter plots. Similarity of methylation levels was assessed using the Pearson correlation coefficient.

Abbreviation	Cell type	Reference
h1	H1 human embryonic stem cells	[6]
h9	H9 human embryonic stem cells	[7]
h9_laurent	H9 human embryonic stem cells	[8]
imr90_ipsc	iPSCs of lung fibroblasts IMR90	[7]
ff_ipsc_19_11	iPSCs of foreskin fibroblasts	[7]
ff_ipsc_19_7	iPSCs of foreskin fibroblasts	[7]
ff_ipsc_6_9	iPSCs of foreskin fibroblasts	[7]
ads_ipsc	iPSCs of adipose-derived stem cells	[7]
h1_bmp4	trophoblasts differentiated from H1 cells	[7]
ff_ipsc_bmp4	trophoblasts differentiated from foreskin fibroblast iPSCs	[7]
ads	adipose-derived stem cells	[7]
ads_adipose	adipocytes differentiated from adipose-derived stem cells	[7]
imr90	fetal lung fibroblasts	[6]
ff	foreskin fibroblast	[7]
hspc	hematopoietic stem and progenitor cells	[9]
cd133hsc	hematopoietic stem and progenitor cells from male umbilical cord blood	[9]
neutrophil	granulocytic neutrophils	[9]
bcell	B cells	[9]

Supplementary Table 1: Methylomes analyzed in this study.

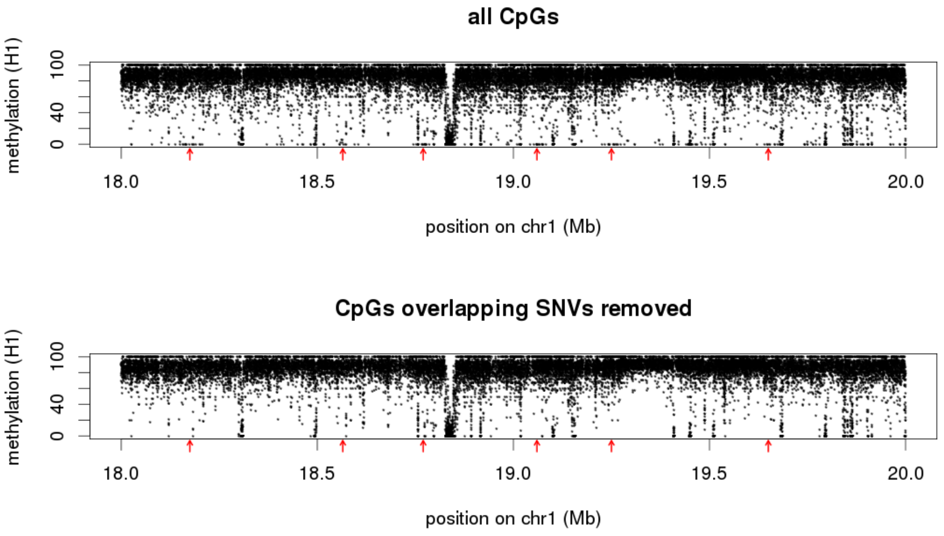

Supplementary Figure 1: Effect of SNVs on methylation levels. Representative example methylation profile for H1, showing 2 megabases of chromosome 1, once with all CpGs (top) and once with all CpGs that do not overlap SNVs (bottom). Falsely called methylation levels due to SNVs are apparent as stretches of unmethylated CpGs that disappear after removal of CpGs overlapping SNVs from dbSNP (indicated by red arrows). Only CpGs with a coverage of at least 5 reads are shown.

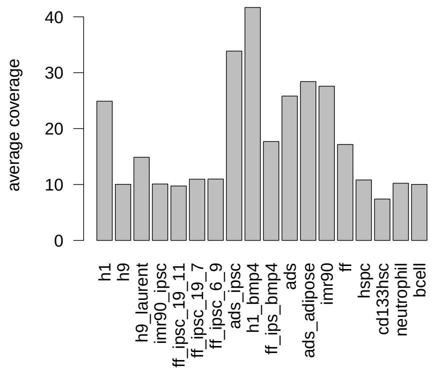

Supplementary Figure 2: Average coverage of the methylomes analyzed in this study. Cell-type abbreviations are explained in **Supplementary Table 1**.

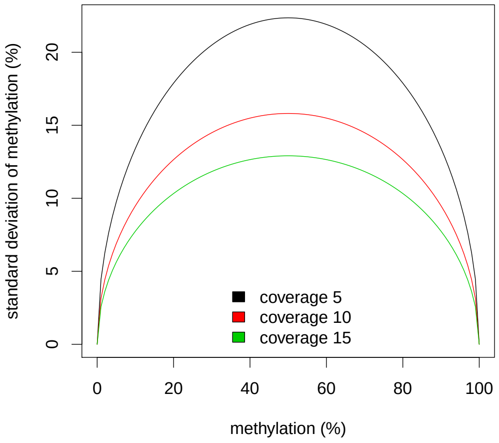

Supplementary Figure 3: Uncertainty in estimation of methylation levels (y-axis) as a function of coverage (indicated in the legend) and the true methylation level (x-axis) assuming binomial sampling.

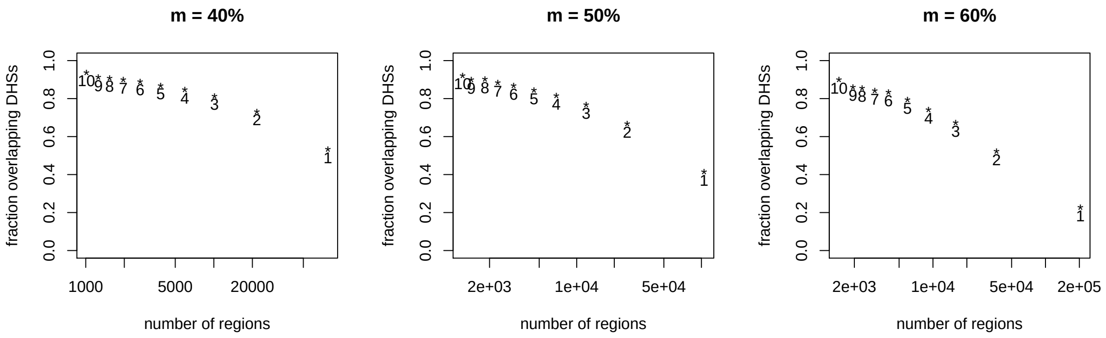

(a) mouse ESCs

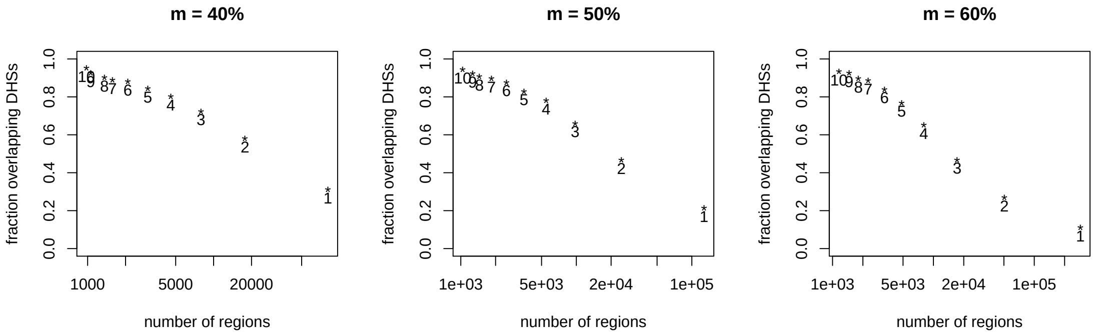

(b) human H1

Supplementary Figure 4: Fraction of regions overlapping DHSs as a function of the cut-off on methylation and the number of hypomethylated CpGs. For both mouse (a) and human H1 (b) ESCs, all CpGs with at least coverage 10 were selected and regions of consecutive CpGs with methylation levels below a given cut-off m (indicated on top of each figure) were identified. Each dot represents all regions containing a fixed number of CpGs (indicated below each dot). The fraction of regions not overlapping DHSs is substantial for regions with only 1 or 2 CpGs and decreases quickly with increasing number of CpGs.

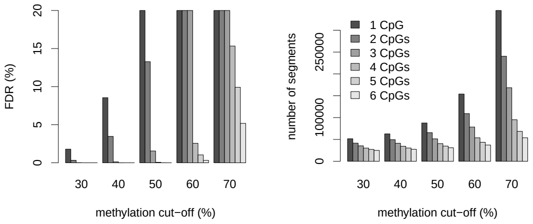

(a) h1

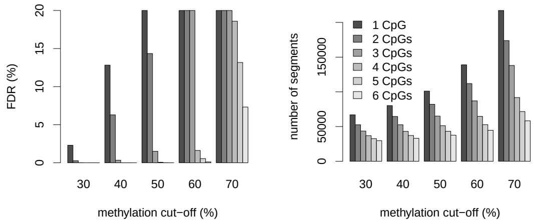

(b) h1_bmp4

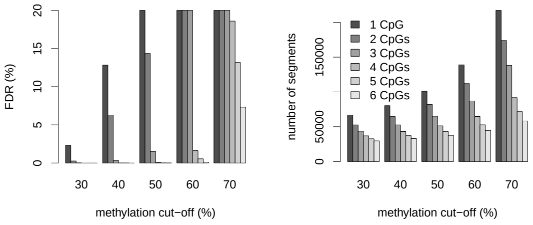

(c) imr90

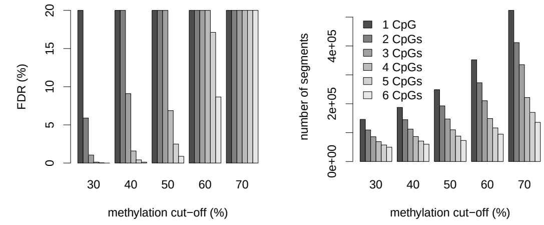

(d) ads_adipose

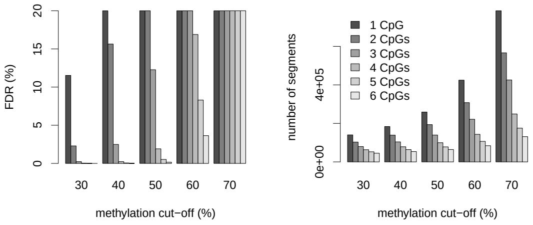

(e) hspc

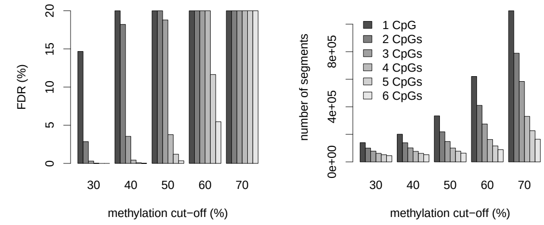

(f) bcell

Supplementary Figure 5: Relationship between false discovery rate (FDR, left), the number of identified regions (right), the cut-off on methylation (x-axes) and the cut-off on the minimal number of CpGs per region (indicated as different shades of grey) for a representative set of human methylomes. The y-axis is limited to reasonably small FDR values (0-20%) and larger values have been set to 20%. Cell-type abbreviations are explained in **Supplementary Table 1**.

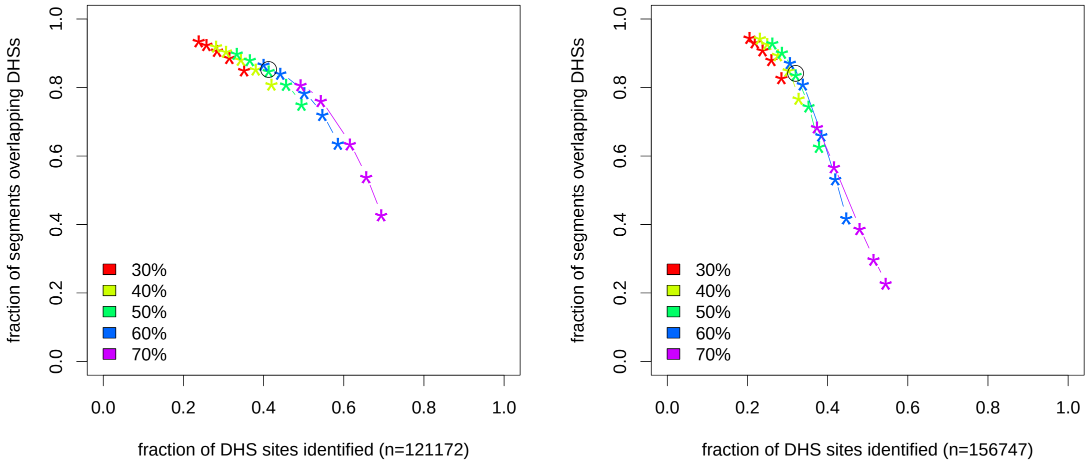

(a) mouse ESCs

(b) human H1

Supplementary Figure 6: Overlap of hypomethylated regions with DHSs as a function of both the methylation cutoff m and the minimal number of CpGs per region n for both mouse (left) and human H1 (right) ESCs. For a given m (indicated in the legend), the fraction of regions overlapping DHSs by at least one nucleotide (y axis) and the fraction of identified DHSs were determined (x axis). The cut-off on n increases, for each m, from right to left, from 1 CpG to 5 CpGs. The parameters chosen in this study for these cell types ($m = 50\%$ and $n = 3$) are highlighted by black circles.

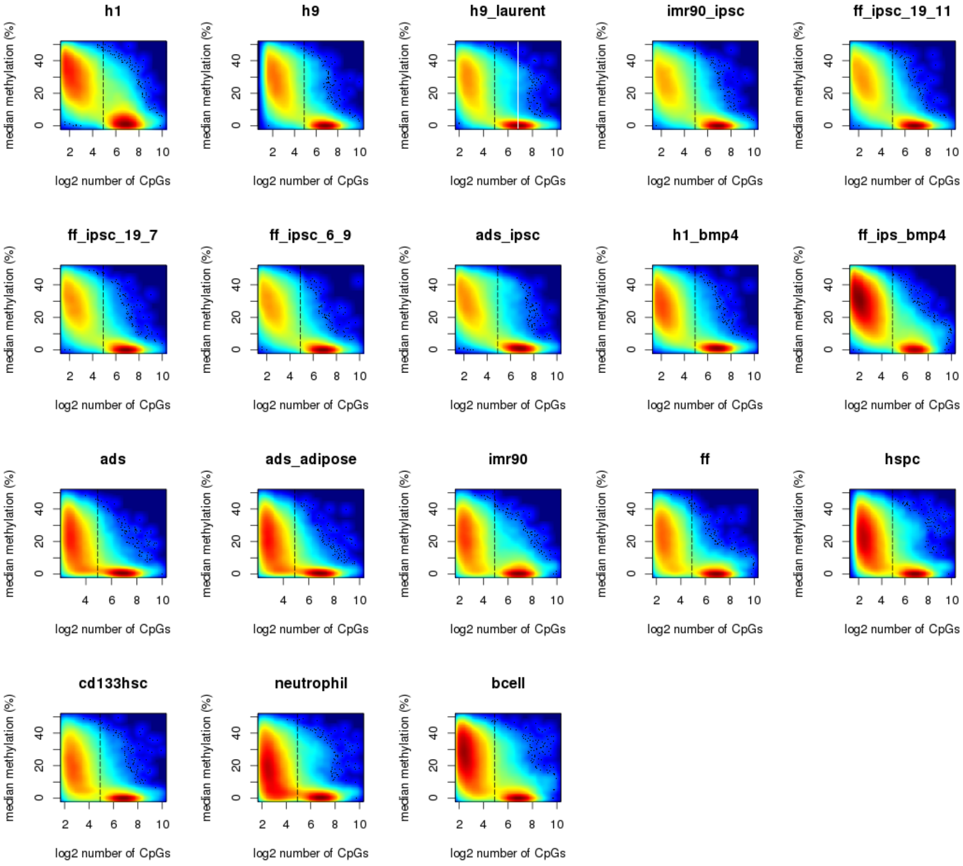

Supplementary Figure 7: Number of CpGs versus median methylation levels for all identified regions and all human methylomes analyzed in this study. The dashed line is at 30 CpGs and represents the cut-off used to classify LMRs and UMRs. The number of CpGs within each region was calculated as the number of CpGs in the genome (and not the number of CpGs covered by at least 5 reads as this number can, for the same region, vary substantially from methylome to methylome). Cell-type abbreviations are explained in **Supplementary Table 1**.

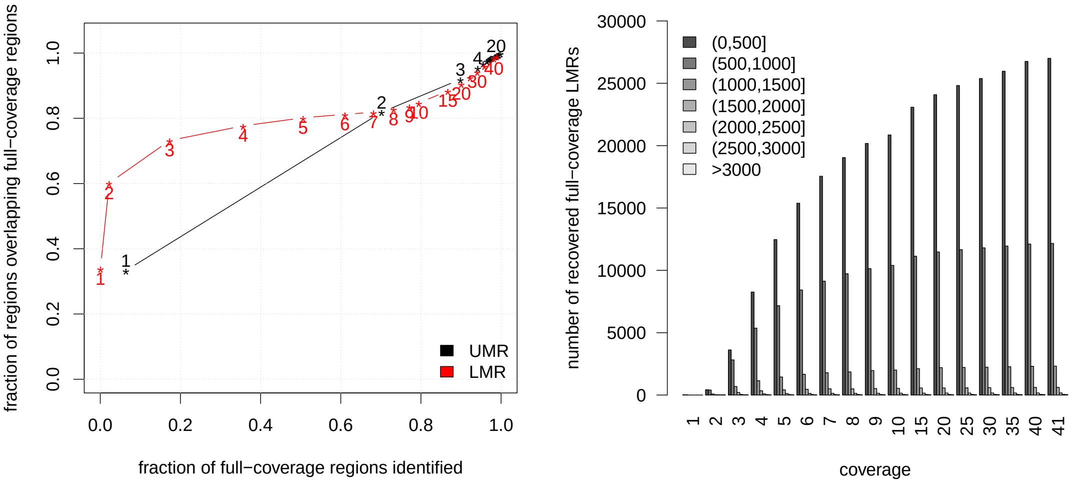

(a) h1_bmp4

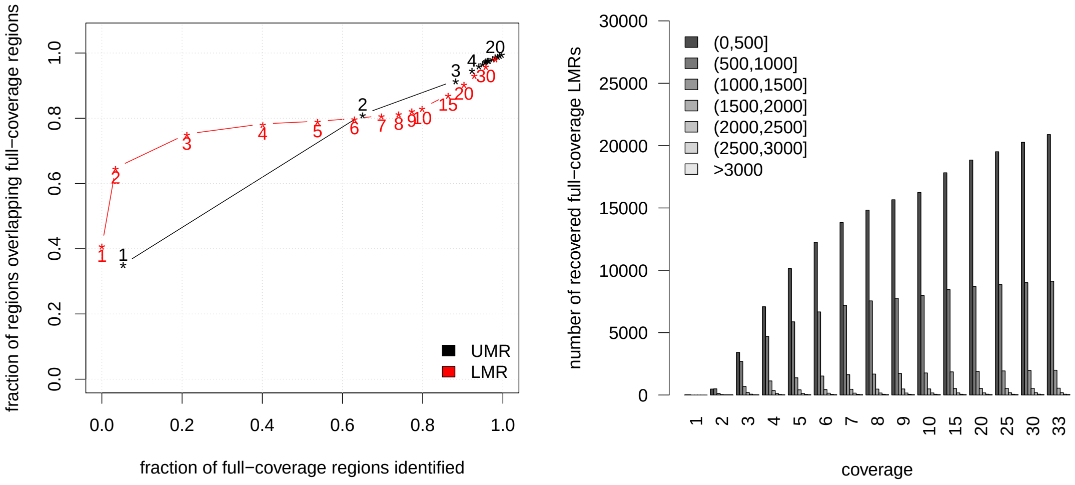

(b) ads_ipsc

Supplementary Figure 8: Coverage requirements to identify UMRs and LMRs. To investigate to what extent the ability to identify UMRs and LMRs depends on the average coverage of a methylome, we subsampled the two highest coverage methylomes (h1_bmp4 and ads_ipsc) at varying coverages from 1 to the maximal coverage (in steps of 1 from 1 to 10 and then in steps of 5) and identified hypomethylated regions using MethylSeekR (see supplementary text for the details of the sampling procedure). The left panels show the number of identified regions divided by the number of regions identified in the full-coverage (ie original) methylome (sensitivity, x-axis) versus the fraction of regions that overlap the regions identified in the full-coverage methylome (accuracy, y-axis). The right panels show the number of LMRs of the full-coverage methylome identified as a function of their length (nts, indicated in the legend) and the methylome coverage (x-axis). Cell-type abbreviations are explained in **Supplementary Table 1**.

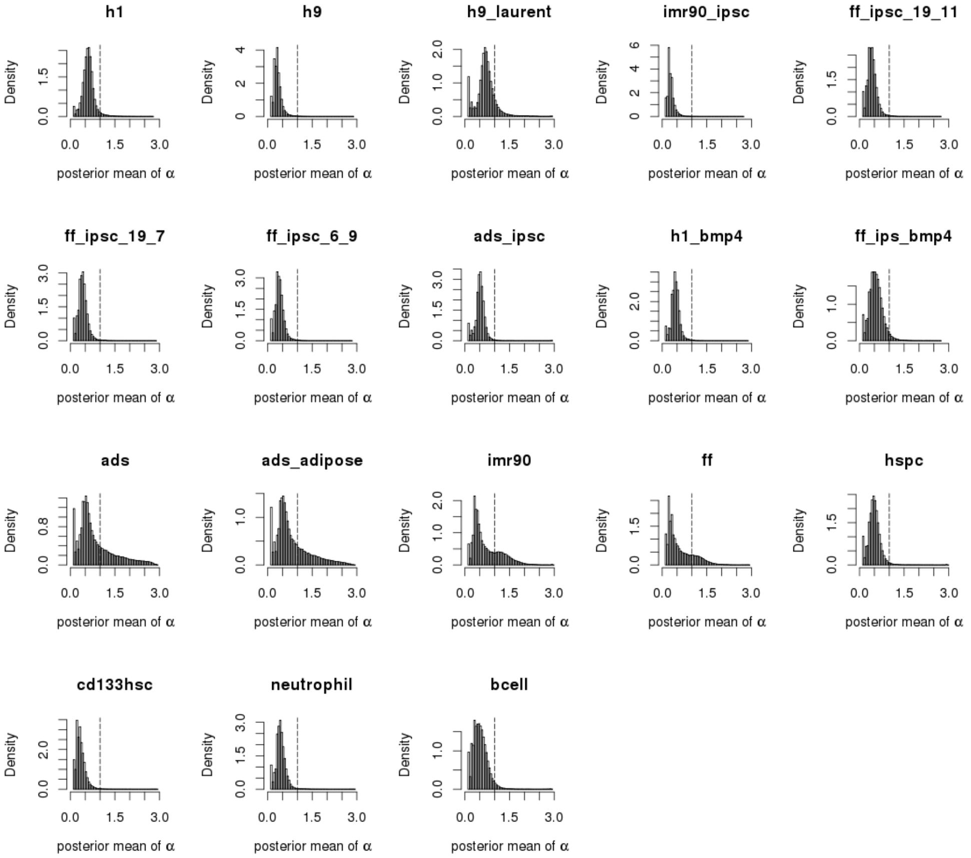

Supplementary Figure 9: The distribution of α-values, which characterize the distribution of methylation levels in sliding windows of 101 CpGs, for all methylomes (Supplementary text). The dashed line lies at $\alpha = 1$, indicating a uniform distribution. Whereas the majority of methylomes have a unimodal distribution with most α below 1 (polarized distribution favoring methylation levels close to 0 and 100% methylation), *ads* and *ads_adipose* have long-tailed distributions and *imr90*, *ff* have a bimodal distribution with a large number of windows with $\alpha \geq 1$ (uniform distribution or distribution favoring intermediate methylation levels), indicative of the presence of PMDs. Cell-type abbreviations are explained in **Supplementary Table 1**. α-value distributions were calculated on chromosome 22.

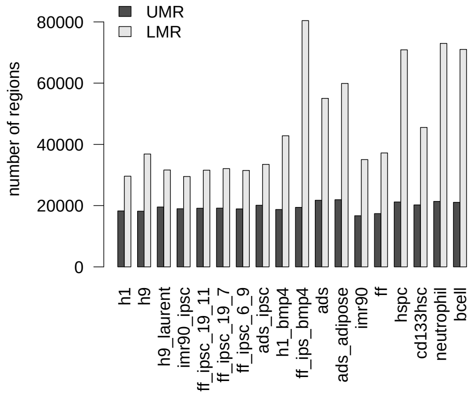

Supplementary Figure 10: Number of UMRs and LMRs identified in each methylome. Cell-type abbreviations are explained in **Supplementary Table 1**.

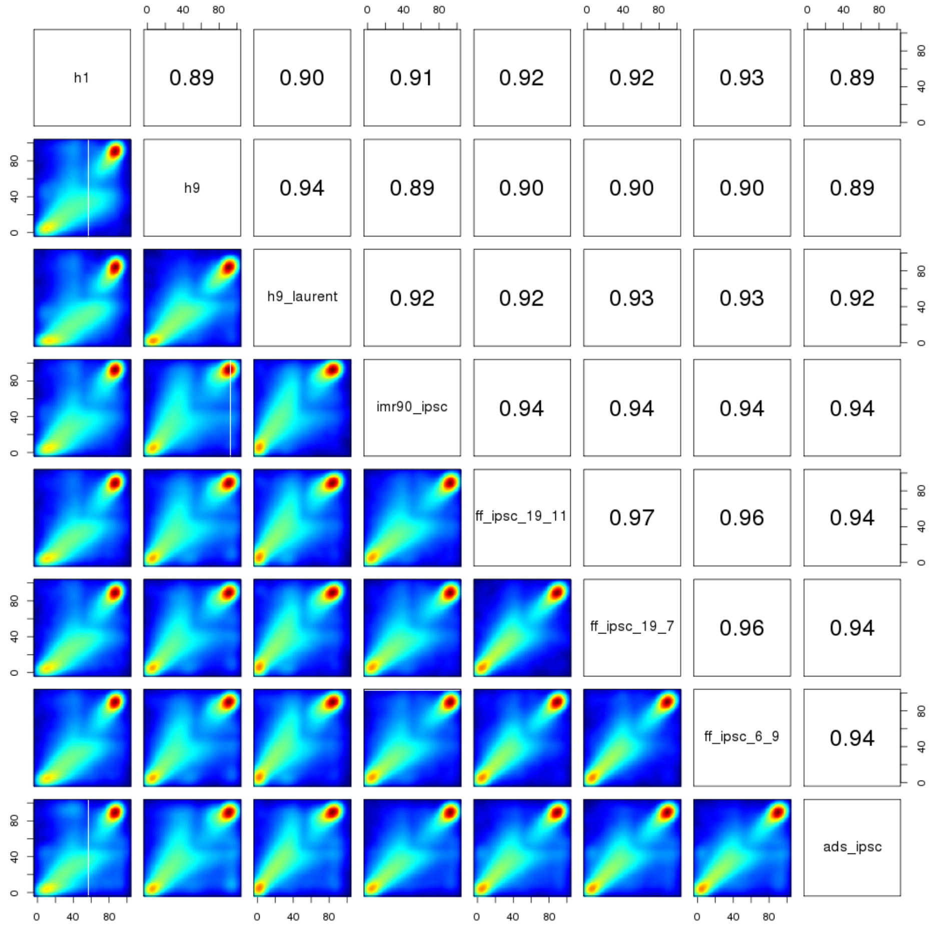

Supplementary Figure 11: Scatter-plot of methylation levels in UMRs, LMRs and fully methylated regions in all pluripotent cells (ESCs and iPS cells). Fully methylated regions were defined as all regions lying between hypomethylated regions (UMRs and/or LMRs). The figure shows good reproducibility between different ESC methylomes (first three samples) as well as between ESC and iPS cell methylomes (remaining samples). The numbers indicate the Pearson correlation coefficient. Cell-type abbreviations are explained in **Supplementary Table 1**.

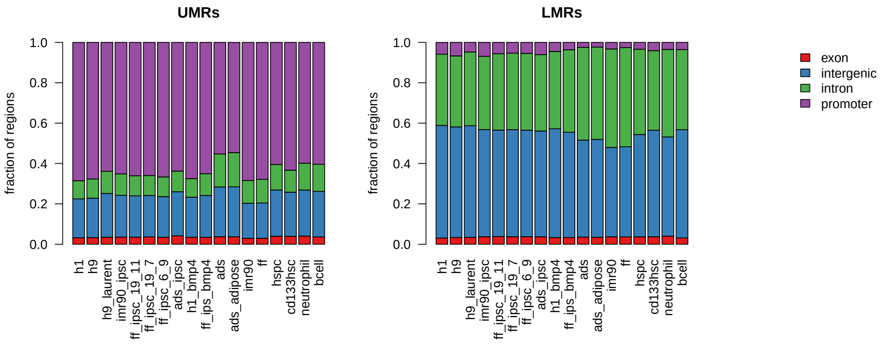

Supplementary Figure 12: Fraction of UMRs (left) and LMRs (right) overlapping different genomic regions. Most UMRs overlap promoters whereas LMRs lie mostly in introns and intergenic regions. Cell-type abbreviations are explained in **Supplementary Table 1**.

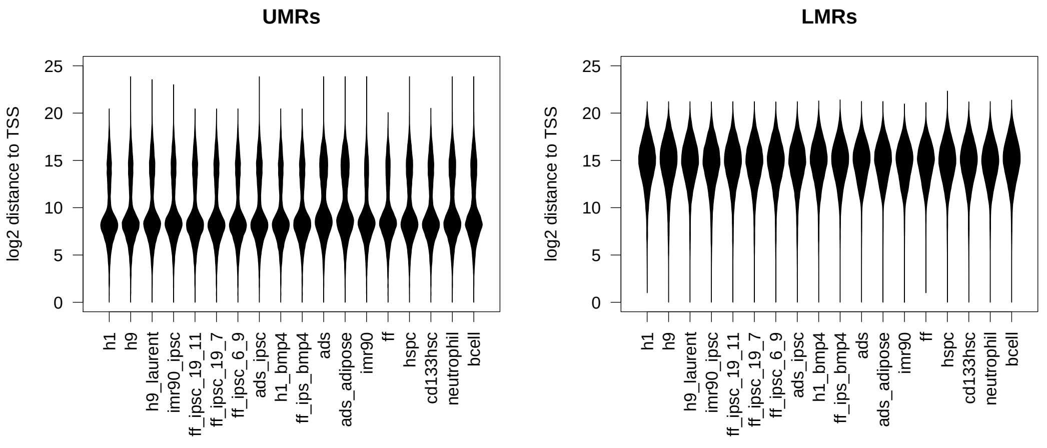

Supplementary Figure 13: Violin plot of the distribution of distances to the closest transcription start site for UMRs (left) and for LMRs (right). Most UMRs are promoter-proximal whereas almost all LMRs are distal to transcription start sites. Cell-type abbreviations are explained in **Supplementary Table 1**.

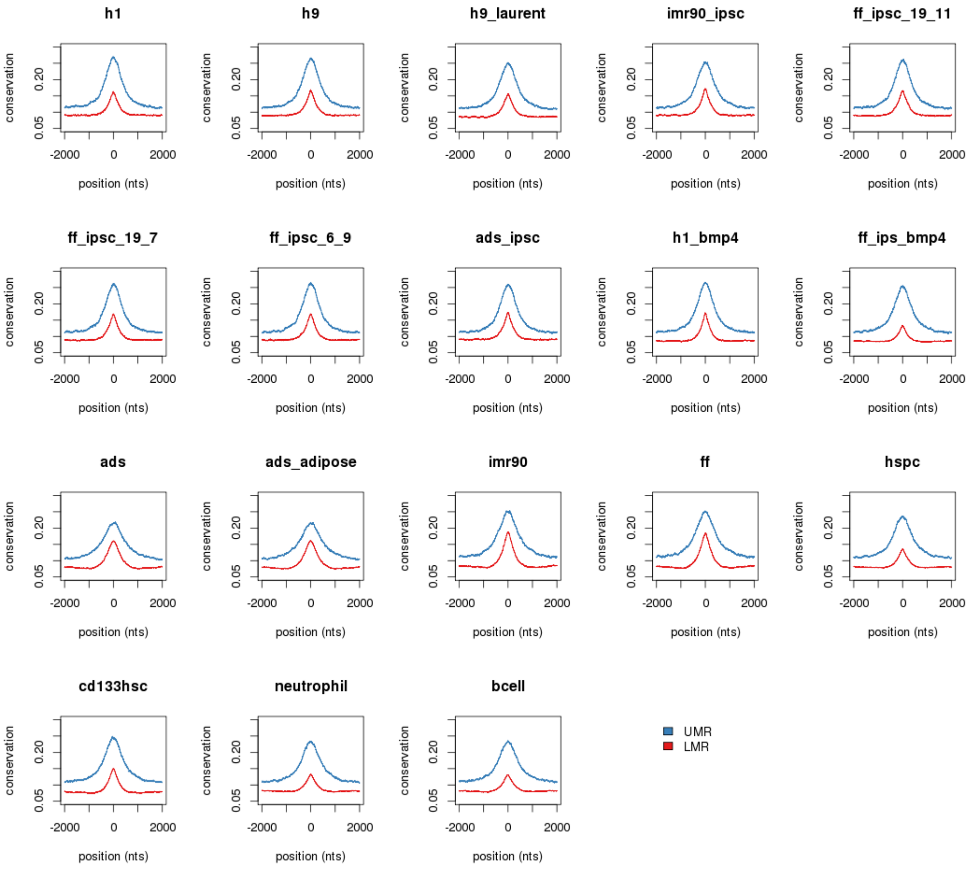

Supplementary Figure 14: Average conservation of UMRs and LMRs. For each methylome, all UMRs and LMRs were anchored at their midpoint and average PhastCons conservation scores per position were calculated. In all cases, both UMRs and LMRs are more conserved than their surrounding regions. Cell-type abbreviations are explained in **Supplementary Table 1**.

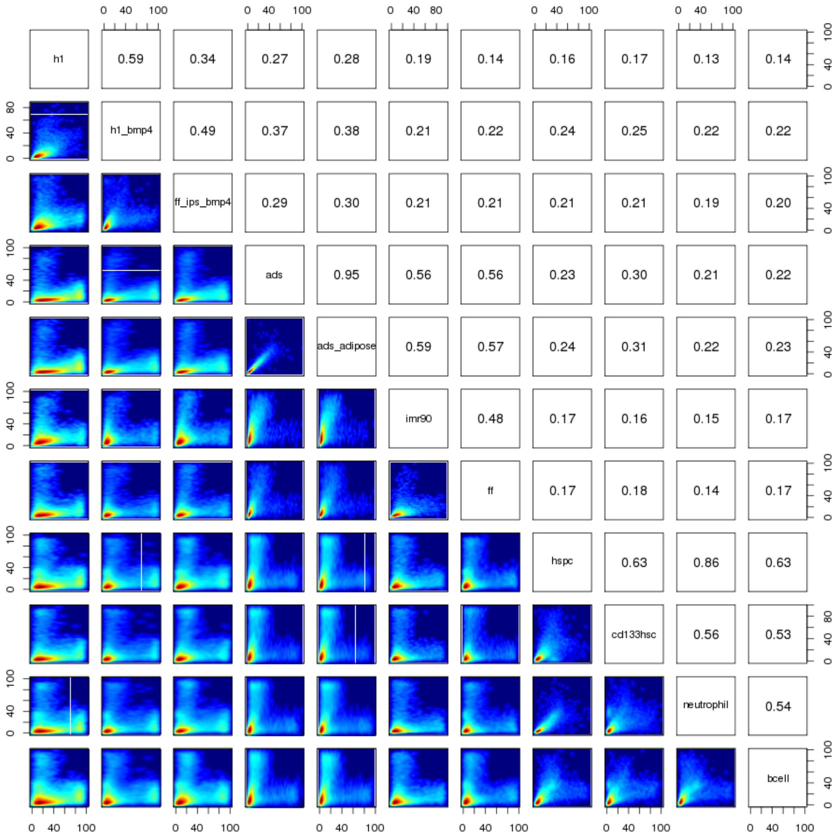

Supplementary Figure 15: Scatter plots of methylation levels in UMRs. For each pair of methylomes, average methylation levels were calculated on the joint set of regions. For pluripotent cells, only one representative sample is shown (h1). UMR methylation levels do not change substantially between methylomes. The numbers indicate the Pearson correlation coefficient. Cell-type abbreviations are explained in **Supplementary Table 1**.

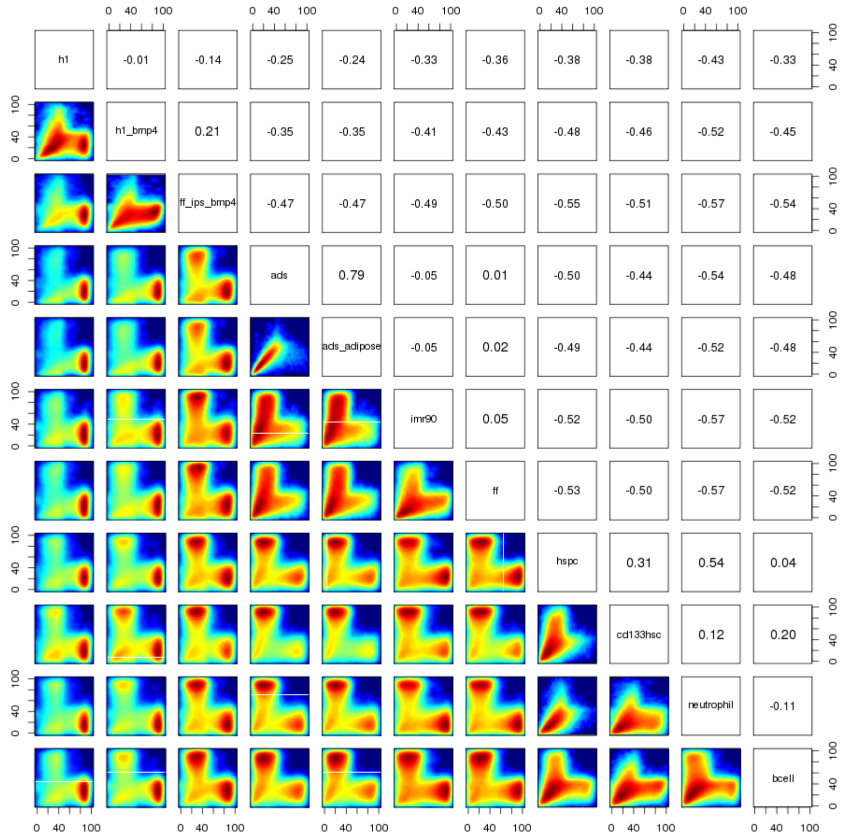

Supplementary Figure 16: Scatter plots of methylation levels in LMRs. For each pair of methylomes, average methylation levels were calculated on the joint set of regions. For pluripotent cells, only one representative sample is shown (h1) . LMR methylation levels are highly dynamic between methylomes. The numbers indicate the Pearson correlation coefficient. For comparisons with PMD-containing methylomes, only regions that do not overlap with PMDs are shown. Cell-type abbreviations are explained in **Supplementary Table 1**.

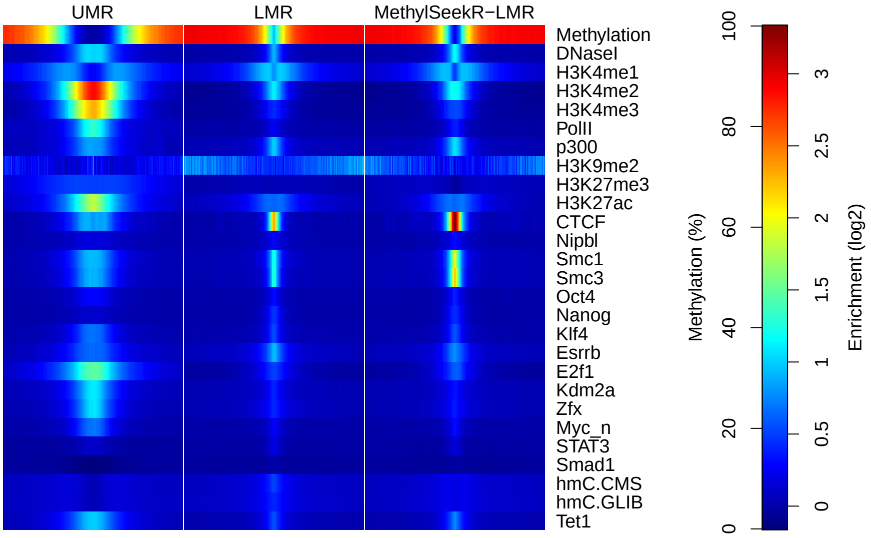

Supplementary Figure 17: Enrichment of chromatin marks and TF binding sites in hypomethylated regions in mouse embryonic stem cells as identified by our previously proposed HMM [1] or MethylSeekR. The main difference between the two methods is that MethylSeekR reclassifies a set of UMRs, as identified by the HMM, as LMRs (Supplementary text). We thus separated the identified hypomethylated regions into UMRs (UMRs according to MethylSeekR), LMR (LMRs according to both the HMM and MethylSeekR) and MethylSeekR-LMR (LMRs according to MethylSeekR, but UMRs according to the HMM approach). Whereas the first set of regions ("UMR") is occupied by PolII and show enrichments for chromatin marks of active promoters (H3K4me3 and no H3K4me1), both the second ("LMR") and third set ("MethylSeekR-LMR") display the characteristics of active distal regulatory regions (enrichments for p300 and H3K4me1, no H3K4me3, no PolII), supporting the classification implemented in MethylSeekR. For details regarding the calculation of enrichments see [1].

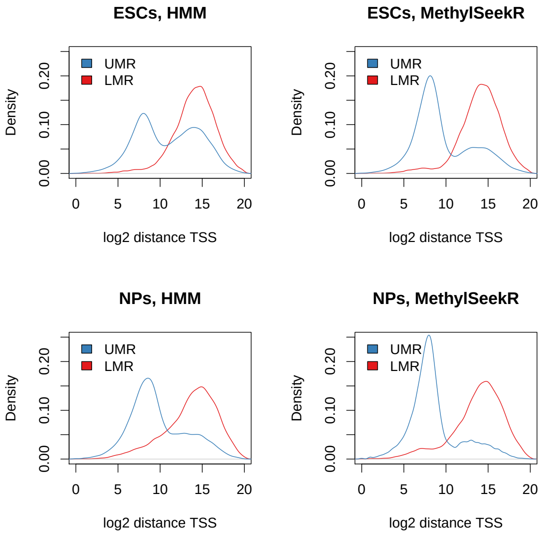

Supplementary Figure 18: Distribution of distances of UMRs and LMRs to transcript start sites (TSS), comparing the HMM segmentation (ESCs, HMM and NPs, HMM) to MethylSeekR (ESCs, MethylSeekR and NPs, MethylSeekR) in mouse embryonic stem cells (ESCs) and neural progenitors (NPs). The classification into UMRs and LMRs used by MethylSeekR provides a much clearer separation of TSS-proximal and TSS-distal regions.

References

[1] Michael B Stadler, Rabih Murr, Lukas Burger, Robert Ivanek, Florian Lienert, Anne Schöler, Erik van Nimwegen, Christiane Wirbelauer, Edward J Oakeley, Dimos Gaidatzis, Vijay K Tiwari, and Dirk Schübeler. DNA-binding factors shape the mouse methylome at distal regulatory regions. *Nature*, 480(7378):490–5, Dec 2011.

[2] S T Sherry, M H Ward, M Kholodov, J Baker, L Phan, E M Smigielski, and K Sirotkin. dbSNP: the NCBI database of genetic variation. *Nucleic Acids Res*, 29(1):308–11, Jan 2001.

[3] Laurence R Meyer, Ann S Zweig, Angie S Hinrichs, Donna Karolchik, Robert M Kuhn, Matthew Wong, Cricket A Sloan, Kate R Rosenbloom, Greg Roe, Brooke Rhead, Brian J Raney, Andy Pohl, Venkat S Malladi, Chin H Li, Brian T Lee, Katrina Learned, Vanessa Kirkup, Fan Hsu, Steve Heitner, Rachel A Harte, Maximilian Haeussler, Luvina Guruvadoo, Mary Goldman, Belinda M Giardine, Pauline A Fujita, Timothy R Dreszer, Mark Diekhans, Melissa S Cline, Hiram Clawson, Galt P Barber, David Haussler, and W James Kent. The UCSC Genome Browser database: extensions and updates 2013. *Nucleic Acids Res*, 41(Database issue):D64–9, Jan 2013.

[4] Birney et al. Identification and analysis of functional elements in 1% of the human genome by the ENCODE pilot project. *Nature*, 447(7146):799–816, Jun 2007.

[5] Michael Lawrence, Robert Gentleman, and Vincent Carey. rtracklayer: an R package for interfacing with genome browsers. *Bioinformatics*, 25(14):1841–2, Jul 2009.

[6] Ryan Lister, Mattia Pelizzola, Robert H Dowen, R David Hawkins, Gary Hon, Julian Tonti-Filippini, Joseph R Nery, Leonard Lee, Zhen Ye, Que-Minh Ngo, Lee Edsall, Jessica Antosiewicz-Bourget, Ron Stewart, Victor Ruotti, A Harvey Millar, James A Thomson, Bing Ren, and Joseph R Ecker. Human DNA methylomes at base resolution show widespread epigenomic differences. *Nature*, 462(7271):315–22, Nov 2009.

[7] Ryan Lister, Mattia Pelizzola, Yasuyuki S Kida, R David Hawkins, Joseph R Nery, Gary Hon, Jessica Antosiewicz-Bourget, Ronan O'Malley, Rosa Castanon, Sarit Klugman, Michael Downes, Ruth Yu, Ron Stewart, Bing Ren, James A Thomson, Ronald M Evans, and Joseph R Ecker. Hotspots of aberrant epigenomic reprogramming in human induced pluripotent stem cells. *Nature*, 471(7336):68–73, Mar 2011.

[8] Louise Laurent, Eleanor Wong, Guoliang Li, Tien Huynh, Aristotelis Tsirigos, Chin Thing Ong, Hwee Meng Low, Ken Wing Kin Sung, Isidore Rigoutsos, Jeanne Loring, and Chia-Lin Wei. Dynamic changes in the human methylome during differentiation. *Genome Res*, 20(3):320–31, Mar 2010.

[9] Emily Hodges, Antoine Molaro, Camila O Dos Santos, Pramod Thekkat, Qiang Song, Philip J Uren, Jin Park, Jason Butler, Shahin Rafii, W Richard McCombie, Andrew D Smith, and Gregory J Hannon. Directional DNA methylation changes and complex intermediate states accompany lineage specificity in the adult hematopoietic compartment. *Mol Cell*, 44(1):17–28, Oct 2011.
